# Does Carbon Footprint Play a Relevant Role in Food Consumer Behaviour? A Focus on Spanish Beef

**DOI:** 10.3390/foods11233899

**Published:** 2022-12-02

**Authors:** Olda Lami, Francisco J. Mesías, Celia Balas, Carlos Díaz-Caro, Miguel Escribano, Andrés Horrillo

**Affiliations:** 1 Department Economics, Universidad de Extremadura, Avda. Adolfo Suárez, s/n, 06007 Badajoz, Spain; 2 Research Institute of Agricultural Resources (INURA), Universidad de Extremadura, Avda. Adolfo Suárez, s/n, 06007 Badajoz, Spain; 3 Department ofAccounting and Finance, Universidad de Extremadura, Avda. de la Universidad, s/n, 10071 Cáceres, Spain; 4 Department Animal Production and Food Science, Universidad de Extremadura, Avda. de la Universidad, s/n, 10071 Cáceres, Spain

**Keywords:** choice experiment, carbon footprint, beef, consumer preference, willingness to pay

## Abstract

Carbon footprint has become a reference indicator of the environmental impact of food production. Governments are increasingly demanding a trend towards low-carbon-footprint production in order to reduce the impact on climate change. In this sense, the study of consumers’ preferences and assessment of products from the perspective of their carbon footprint is crucial to achieve a green and circular economy. This paper specifically attempted to assess consumer preference and willingness to pay for beef that has been carbon-footprint-labelled as an attribute. In order to attain this objective, a choice experiment was designed and applied to a total of 362 Spanish consumers. The results revealed a positive preference towards beef produced in low-carbon-footprint systems. In addition, the segmentation of these consumers revealed the existence of a group of consumers who prioritise environmental impact over product price.

## 1. Introduction

Greenhouse gas (GHG) emissions have increased in the last century [1] on account of the growth of the world’s population and the change towards more intensive production and consumption models. Such an increase in GHGs has contributed to current climate change, where the agricultural sector and food production are playing an important role [2]. In this context, animal food products with production models that involve an excessive use of resources, fossil energies, and, definitely, the generation of a significant environmental impact are not considered sustainable. In fact, the livestock industry is responsible for 14.5% of the GHG emissions [3].

FAO’s publication of works [3,4] highlights the role of enteric emissions from ruminants in the impact on global warming. In such publications, ruminants are pointed at as one of the main players in the generation of man-originated GHGs. The message these reports have transmitted has not only harmed the reputation of the animal production industry, but especially, that of pasture-based animal production systems. 

However, other research has questioned the aforementioned reports [5,6]. For example, studies on global warming have stated that the use of various techniques in ruminant feeding can reduce the enteric emission of GHGs [7,8]. Other studies have also pointed out how extensive livestock production systems can sequestrate carbon into the soil [2,9].

In this sense, the European Union (EU) believes that the kind of production model has a clear impact on the level of GHG emissions. In fact, one of the EU proposals aimed at mitigating the increase in GHG emissions is to meet the target for 25% of the agricultural areas of the EU to have moved to organic production by 2030. Together with this target, the proposal also hopes to attain the reduction in fertilisers and harmful pesticides, as well as achieving the goal of 30% of the European areas implementing systems for environmental protection [10]. All these proposals open a new future where less intensive and more sustainable production systems can help improve the situation of livestock production [11].

On the other hand, the lack of market discrimination of livestock production systems has raised a general concern about the consumption of meat products, regardless of their origin or production model. This, in turn, has led to an increase in the search for products that are alternative to meat. 

This has driven the food industry to begin the development of new food products with additional benefits to consumer health [12]. Products such as “meat substitutes” (products made from alternative protein sources such as vegetables, algae, insects, and mycobacteria) and “cultured meat”, which attempt to offer alternatives to a number of consumer preferences [13], have recently entered the market. However, not all the consumers from western countries are willing to reduce their meat consumption [14,15,16,17].

In order to reach a target with lower emissions, a commitment from both the industry and consumers is needed. Food industries can adopt different strategies to reduce their carbon footprint, although some of them may affect consumers and, therefore, may not be well accepted. For example, the production of foods with higher emission levels, such as beef, can be reduced and replaced by less polluting ones, for example, pork or chicken. In addition, supply networks could be redesigned to reduce imports of raw materials, which could be sourced from nearby markets, and to optimise delivery policies [18,19]. Moreover, reducing food waste is an essential strategy [20], not only because agrosystems are not forced to produce additional food, but also to avoid the extra consumption of energy and inputs (packaging, labelling, etc.) and waste disposal [18]. 

In the case of consumers, the way they can reduce climate change is by adopting a more environmentally friendly lifestyle [21] that is more in accordance with sustainable development objectives [22]. In this sense, food consumption is a major field for action [23,24] and the implementation of carbon footprint (CF) labelling systems is an option for consumers to become environmentally friendly [25]. CF labelling can be defined as “a measure to inform of the total carbon dioxide emissions to include other GHGs such as nitrous oxide and methane” [26], which is currently used by numerous companies worldwide in food production as well as in other industries. Recent research shows how consumers are generally in favour of this kind of labelling [27,28]. 

Nevertheless, the importance of carbon labelling is limited, as labels are not affixed to all products and they are very varied. Additionally, the results of some research projects differ in terms of how willing consumers are to pay or not for this type of labelling [25,28,29,30,31,32,33].

On the other hand, the information in these labels is not easily interpreted and causes confusion to consumers, making it hard for them to make adequate decisions and change their consumption habits [27]. In addition to this fact, the calculation method for the CF is neither unique nor popularly known by consumers, which also causes controversy [25,34]. There are also studies that reveal how consumers prefer simple and precise, scale-type, colourful CF labels, with an absolute CO_2_ eq. number per unit that helps identify food products that are environmentally friendly [28,35,36,37,38].

Although there are already studies dealing with the role of CF labelling on consumer food behaviour and preference, such as those of [39,40,41,42], the purpose of this particular piece of research is to complement them by dealing with the analysis of the CF concept and other environment-related labels that exist in the market and how these can impact consumer purchase behaviour and choices.

Specifically, beef was chosen for this study because it is a type of meat that is widely consumed in Spain and whose consumption has been declining steadily for more than a decade. Even so, it is the third most consumed type of meat in Spain, only behind chicken and pork [43].

## 2. Materials and Methods

### 2.1. Data Collection

The data were collected in January and February 2021 through an online questionnaire using Google Forms. This way of obtaining the data presents a series of benefits such as flexibility, time-saving in the collection of answers, and error elimination when introducing the data [44,45], which has led to an increase in its use for research purposes. 

The questionnaire used is presented in Table S1 in the electronic supplement and consists of four sections. The first section comprises consumer habits. Consumers were asked about their frequency of consumption, site of the purchase, and the most relevant factors when it came to shopping, amongst other questions. The second section dealt with carbon footprint (CF) in food production and climate change. The third section included a choice experiment (CE) aimed at analysing the impact of CF on consumer preference for beef fillets (an example of the filleted beef used for this study is shown in Figure S1 in the electronic supplement). Finally, the fourth section contained the socioeconomic information. The survey was validated through a test with 10 consumers (not included in the final sample) in order to detect potential errors and difficulties of understanding. 

The participants were contacted by email using databases from previous research studies. They were sent an email with a description of the research, and their collaboration by fulfilling the questionnaire was requested. The link to the questionnaire was included after this message. Due to the project’s budgetary constraints, participants in the study were not financially rewarded, although they were offered the possibility of receiving information regarding the results of the research. The final sample consisted of 362 consumers who sent valid answers and can be considered as a representative sample of the Spanish population in terms of age (the residents in Spain within a range of 19–34 years are approximately 20% of the population; those aged between 35 and 50 account for 28% and those aged more than 50 years represent 52% [46]) and in terms of sex (with 49% of the residents in Spain being males and 51% females [46]), with the maximum error being 4.6% and a 95% confidence interval.

As shown in Table 1, the sample consisted mostly of females, participants younger than 50 years old, with low to medium incomes and university degrees. 

### 2.2. Choice Experiment Design

As stated by [47], stated preferences techniques are recommended when consumers must make choices in situations requiring hypothetical markets. In this sense, CE is an adequate tool for this research. Given its potential, CE has been widely used in the agrifood industry to analyse, for instance, consumers’ preference for beef [48], pork [49], or organic food products [50].

CE is based on the idea that a good or service can be described by its attributes [51], and the fact that consumers make decisions based on such attributes and their various levels [52]. The CE habitually consists of the presentation of various options of the same product containing combinations of the various attributes (and their respective levels) with the purpose of providing consumers with choices to make based on their preferences. An advantage of the CE against all other methods is the fact that it provides the closest reflection of the typical purchase decision of a real market consumer [53], which makes responses easier.

The selected attributes and levels must define the analysed product and reflect the attributes that are most important for the consumer in the course of their purchasing process [54,55]. Specifically, Table 2 shows the selected attributes and levels.

It was considered that the study should be narrowed down to a specific type of meat that the participants could assess more easily. Therefore, the product presented to the participants in the CE was pre-packaged filleted beef (1-kg tray). Beef was chosen not only for being one of the most popular meats in Spain in terms of consumption, but also because it is one of the meats most flagged for its role in climate change [56]. Therefore, improving the sustainability of its production processes could be strongly appreciated by consumers.

The above selection of attributes and levels was made according to the characteristics of the selected product and the previous review of the literature. Specifically, the production system and its levels (intensive vs. extensive) have been widely applied in CE for agrifood products and more specifically in meat products [57,58,59,60].

On the other hand, the information on the origin is an attribute used by consumers in order to identify the product as well as its quality. Specifically, product origin has been seen to have a significant impact on consumer decisions [57,59,61,62,63]. There were three levels selected: meat produced locally, nationally, and imported. 

The CF label is presented as a measure to indicate the set of carbon dioxide emissions caused directly or indirectly during the process of making the product. Given that it was expected for many consumers to have little knowledge of this concept because of its scarce dissemination in the market, the following information, also reported in Table S1, in the questionnaire in the electric supplement was provided to the participants so that they could read it before the CE was conducted:

*“’Carbon footprint’ is an indicator that allows the quantification of greenhouse gas (GHG) emissions that are generated in the course of production of a good or a service, expressed in kilograms of carbon dioxide (CO_2_) equivalent. This is, CF indicates how may kilograms of carbon dioxide equivalents (CO_2e_) are emitted into the atmosphere for every kilogram of product being produced.*

*For example, in livestock production, this indicator will vary depending on the type of farm and livestock management system, with extensive or organic production systems having lower GHG emissions. On the other hand, pasture feed (thus reducing the need to purchase foodstuffs) and lower dependence on transportation (for example, when the production processes are near the area of consumption) make the CF become more reduced.*

*The CF varies with ranges being 9 to 28 kg of CO2e per kg of produced meat. These values will depend on:*

- *The livestock farming and management system*
- *Self-sufficiency of farms in terms of animal feeding (pastures-foodstuffs)*
- *Energy consumption (in terms of transportation, a car has emissions of approximately 0.22 kg of CO_2e_ per kilometre, whereas a truck has an average of 0.66 kg of CO_2e_ per kilometre)*

*For example, organic extensive farms with animal feeding based on pastures and little dependence on the consumption of foodstuffs, situated near the area of consumption, will produce lower CF”.*

The selected levels were low (8 kg of CO_2e_/kg), medium (18 kg of CO_2e_/kg), and high (28 kg of CO_2e_/kg) CF per kg of filleted beef. These values were chosen based on previous studies such as that of [64] where the average carbon footprint for 1 kg of beef in Spain was found to be 18.21 kg. Organic certification was included as an attribute due to its impact on consumer behaviour when purchasing beef [58,60,62,63]. 

Lastly, price is an attribute that tends to be incorporated in CE with the purpose of understanding the willingness to pay for a product and for each of the selected attributes, and therefore, most of the studies added this attribute to the analysis [57,59,62,63]. Specifically, the three price ranges included in this study were selected based on the authors’ observation of retail prices for packaged filleted beef in the Spanish supermarkets: the lowest price (EUR 10/kg) is the price of conventional beef from intensive systems; the second price range (EUR 15/kg) is the average price of beef; lastly, the third price range (EUR 20/kg) is the price of higher-quality beef reared in extensive systems. 

**Figure 1:**
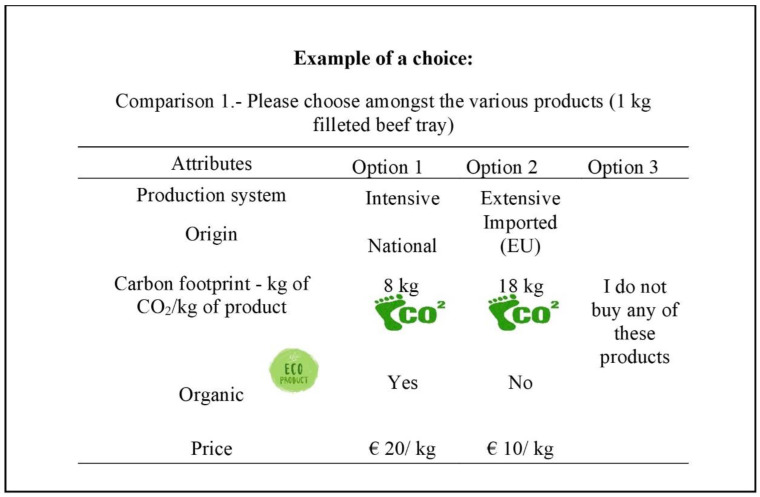
Illustration of an example of a choice set included in the questionnaire.

The total number of hypothetical products that can be created by combining the selected attributes/levels amounts to 108 (2 × 3 × 3 × 2 × 3). Taking into account that they are presented as “choice sets” that are made up of two products plus a “no-purchase” option, there would be a total number of possible comparisons of 11,556 (108 × 107), which is unmanageable in economic and time terms. Therefore, a fractional design was used to reduce the number of comparisons to an efficient level by using Stata’s “Dcreate” module, which allows such designs to be generated [65]. This module uses Fedorov’s modified algorithm to create an efficient design [66]. Finally, six choice sets were created and used for the survey. Figure 1 shows an example of a choice set.

Cheap talk was used to correct the hypothetical bias that can arise in this type of study. Thus, in line with previous research [13], a text explaining the hypothetical bias and its importance in the validity of the study was incorporated into the questionnaire, as shown in Table S1 in the electronic supplement. Finally, participants were asked to try to provide their unbiased answers to the CE, trying to actively put themselves in a real shopping situation.

### 2.3. Conditional Logit

CE is a derivative of random utility models [67], where the utility function of each individual is presumed to be the result of the addition of two terms, a deterministic term that can be described as a function of the factors that have an influence on the utility of individuals, and a non-observed random term that is considered stochastic. Thus, following [68], we can assume that, in a sample of *N* individuals with the option to choose between J alternatives in T occasions, the utility for an individual *n* derived from the choice of alternative *j* on occasion *t* is as follows:

(1) $$U_{njt}=\beta_{n}^{’}x_{njt}+\varepsilon_{njt}$$

where $\beta_{n}^{’}$ is the specific coefficient vector for each individual, $x_{njt}$ is the vector of observable attributes of individual *n* and option *j* on occasion *t,* and $\varepsilon_{njt}$ is the random term we presume to be an independently and identically distributed extreme value. Therefore, the probability that *n* responds by choosing alternative *i* at choice *t* is given by the following expression

(2) $$L_{nit}\left(\beta_{n}\right)=\frac{exp\left(\beta_{n}^{’}x_{nit}\right)}{\sum_{j=1}^{J}exp\left(\beta_{n}^{’}x_{njt}\right)}$$

Expression 2 is the logit conditional formula (McFadden, 1973).

A series of reference values were established, allocating them a “0” utility. The selected reference values were “Intensive” (for attribute “production system”), “imported (EU)” (for “origin”), “Carbon footprint 8 kg” (for “CF”), and “non-organic” (for “organic”). Therefore, the utility $U_{njt}$ obtained by individual *n* from alternative *j* in choice set *t* can be defined as follows:

(3) $$\begin{array}{cc} U_{njt}=\beta_{0}ASC\;+ & \beta_{1}EXT_{njt}+\beta_{2}LOC_{njt}+\beta_{3}NAT_{njt}+\beta_{4}FOOT\_2_{njt}+\beta_{5}FOOT\_3_{njt}+\beta_{6}ORGANIC_{njt}\\ & +\;\beta_{7}PRICE_{njt}+\varepsilon_{njt} \end{array}$$

where ASC is the acronym for the alternative specific constant and represents the non-purchase option among the different options. EXT stands for extensive production, LOC stands for local production, NAT is for national production, FOOT_2 is for carbon footprint 18 kg, FOOT_3 stands for carbon footprint 28 kg, ORGANIC is for organic production, and PRICE is for the value of product/kg.

### 2.4. Willingness to Pay

Willingness to pay for each attribute can be calculated as the price was included in the analysis, and therefore, the monetary assessment consumers make of a specific level of each attribute can be made in comparison to the reference level of the same attribute. Specifically, willingness to pay for a level of an attribute, i.e., $WTP_{k}$, is calculated by dividing the level of that attribute, $\beta_{k}$, by the corresponding price parameter, $\beta_{PRICE}$ with the opposite sign according to the following:

(4) $$WTP_{k}=-\frac{\beta_{k}}{\beta_{PRICE}}\;$$

### 2.5. Consumer Segmentation

For the purposes of this study, it was decided that consumer segmentation should be applied in order to further analyse the preferences of respondents towards CF and the purchase of food products according to their CF. The object of analysis here was to identify different consumer groups in order to reveal varied preferences and behaviours towards the purchase of carbon footprint-labelled food products. The calculations were made using the cluster module of statistical software package IBM SPSS 21, using a two-stage cluster, where an initial hierarchical cluster helped determine the number of clusters, thus refining the results with a final *k-means* cluster. The *inputs* in the analysis were several variables included in the questionnaire, which could characterise the level of awareness, behaviour, and attitudes of the respondents towards CF labelling and its use in food. The final solution selected consisted of three segments.

## 3. Results and Discussion

### 3.1. Choice Model for the Overall Sample

Table 3 presents the aggregate results of the conditional choice model for the overall sample. The sign of the coefficient for each level indicates the utility that the presence of this level adds (positive sign) or detracts (negative sign) to or from the reference level in the consumer’s view. 

The results in Table 3 show that most of the attributes have a positive impact on respondents’ utility. Thus, the results for “Origin” indicate that consumers obtain more utility when choosing beef produced locally or in Spain (as compared with EU-imported beef). A similar situation is that of the “Production system” (extensive being preferred against intensive) or “Organic” production, which have a positive impact on the preferences of respondents compared to their baseline reference levels. Regarding the other attributes under consideration, their negative coefficients indicate a lower preference as their values increase (e.g., the higher the price, the lower the preference). 

The z-value parameter helps obtain the relative importance for each level of the various attributes [69]. For example, the highest z-value corresponds to the local and national levels; therefore, “Origin” is established as the main attribute in the eyes of the consumer. High levels of carbon footprint (CF) followed by an extensive production system are next, and, finally, organic and price are the last attributes.

These results are in line with previous research, such as that of [70] who found that consumers preferred local food products to imported products and [71] who stated that country of origin was one of the main aspects influencing consumer preferences for food. Regarding beef, [72] indicated that origin was the most important piece of information demanded by European consumers, while [57,73] found that origin was one of the most important attributes regarding consumer’s preferences for beef. 

Regarding the attribute “Organic”, [74] found that organic production was very relevant when purchasing meat, while [57,73,75] saw that consumers preferred organic beef to beef produced conventionally. 

Additionally, the negative sign for attribute “Price” is consistent with the habitual behaviour in demand and is in agreement with other research studies on meat preferences [57,76,77]. 

Finally, the coefficients for “CF” portray consumers’ increasing concern about the influence of animal production on the environment through deforestation and GHG emissions, with meat production being markedly criticised in this respect [2]. Our findings reflect that a lower CF is preferred by consumers in accordance with the aforementioned trend. However, [73] found that CF was not significant in terms of consumer preference, which was associated with consumers’ lack of familiarity with the CF concept and logo, whereas [78] found that CF had a moderate impact on consumers’ meat choices.

### 3.2. Characterisation of Consumer Segments

Table 4 describes the clusters based on different variables related to consumers’ attitudes and behaviour towards meat purchasing and CF.

The values in Table 4 show significant differences amongst the three clusters. Cluster 1 shows average values above the other two clusters for all the questions, this is, it gives greater importance to factors such as price, packaging, label quality, origin, place of purchase, or even the meat production system used. Cluster 2 shows average values situated between those of cluster 1 and cluster 3, implying that this type of consumer gives scores that are closer to the overall sample. Finally, Cluster 3 grants scores well below the overall average, although consumers in this cluster are the most frequent meat consumers. 

CF awareness is higher in Cluster 1 than Cluster 3, with Cluster 2 showing an intermediate level, although all the respondents state that they are aware of the CF to a medium-high level. Finally, and in terms of willingness to pay, this is higher in Cluster 1, which is in line with the group’s characteristics. The level of familiarity with the CF logos is very low, with nearly most consumers not being familiarised with them. 

In terms of the sociodemographic variables, only sex presents significant differences amongst clusters, as Cluster 1 is mainly made of women (63%), whereas Cluster 2 is balanced in terms of sex, and Cluster 3 presents a higher number of men (59%). As different authors argue [79,80], sociodemographic characteristics have lost a great deal of their capacity to explain consumer groups. Thus, [81] proposed the use of other variables, such as lifestyles, in segmentation studies. For this reason, and to complete the overview of the consumer segments, Table 5 presents a description of their lifestyles and attitudes towards the environment, as deduced from the answers to question no. 24 (see Table S1), against the same description for the overall sample.

As Table 5 shows, all the variables listed present statistically significant differences. In terms of cluster differences, Cluster 1 shows average values on attitudes and lifestyles that are above the other two groups. In this sense, Cluster 2 is the consumer group with average scores that are closer to the overall sample. The last cluster shows scores below the overall sample and below the other clusters for almost all the questions. 

On the basis of the consumer behavioural characteristics, lifestyles, and environmental attitude described above, it is possible to establish a definition of each of the three clusters:

The first cluster consists of respondents with a tendency towards establishing higher scores to purchasing characteristics and showing higher scores for environmental attitudes. This type of consumer could be defined as “Conscious consumers”. In terms of Cluster 2, the levels for each of the above variables are intermediary and closer to the overall sample, which gives this consumer group the denomination of “Balanced consumers”. Finally, Cluster 3 reveals lower scores for purchasing factors, and relatively low scores for environmental attitudes and purchasing behaviour, which reflects these consumer trends towards the “Indifferent” consumer. 

### 3.3. Choice Experiment Per Segment

CE has been subsequently applied to each cluster in order to delve into the consumers’ preference patterns. Table 6 presents the results of the CE for each cluster.

In terms of segmentation, Cluster 1 shows a non-significant coefficient for price as well as for “Footprint 18 kg”, whereas the remaining coefficients are similar to the overall sample. Cluster 2 reveals coefficients both in terms of sign and significance, which are very similar to those obtained in the overall sample. Finally, Cluster 3 shows more differentiated results compared to the other two groups and the overall sample. Additionally, it shows coefficients with similar significance to those obtained for the overall sample, but their importance is higher for price and lower for organic coefficient. 

A final issue that is relevant for the research is consumer’s willingness to pay. Thus, Table 7 shows the willingness to pay for each of the significant attribute levels in comparison to the base level, both for the overall sample and the various clusters. 

Table 7 shows that, generally, consumers are more willing to pay for attribute “origin”, mainly for “local” followed by “national” levels. It is also noteworthy that the different levels of the CF attribute show negative willingness to pay, which becomes higher as the CF increases. Next is production system, which is situated in the average level in terms of willingness to pay amongst the various levels of CF. Finally, attribute “organic” is last in terms of willingness to pay. 

In terms of willingness to pay according to cluster, the most outstanding is Cluster 1 with no values, as coefficient “price” is not significant. Cluster 2, on the other hand, reveals a higher willingness to pay than the rest of the overall sample. Finally, Cluster 3 presents lower willingness to pay.

The results reveal how willingness to pay is closely associated to the consumer characteristics and profile. In the case of environmental labelling, such as CF, consumers appear to be sensitised, as they score negatively in price for GHG emissions of a certain type of meat. This proves consumers’ increasing interest in production methods and the environment and the effect of food on health [82], which has also caused a wave of debate in the media [83]. Thus, environmental threats such as global warming or air and water pollution have become social challenges, which are becoming more and more urgent [84]. This has led to a significant increase in the level of awareness of the institutions, businesses, and individuals of the impact of human activities on the environment in the latest years [73,85]. 

In this sense, CF as an indicator of GHG emissions has become more and more popular due to social concern for the reduction in emissions in order to mitigate climate change and be a reference for assessment [86], as well as measuring the impact on the natural resources and climate change [87,88].

In terms of origin, and especially in relation to its “local” level, the willingness to pay found in this study is no doubt closely linked to the reduction in GHG emissions. As a result, the production and marketing of proximity or local foodstuffs is increasing steadily. Apart from the environmental factor, the concept of proximity is associated with the positive economic and social development effects it provides, which are becoming more and more important for consumers [63,89,90]. 

It is important to point out that for willingness to pay for certain environmental attributes, it is necessary to have adequate and comprehensible labelling to allow consumers to consider environmental impact [33]. Thus, the lack of product information can be a barrier to prevent certain segments from selecting more environmentally friendly products [91]. This is added to the fact that on many occasions, the consumer does not understand the meaning of labels such as the CF label [28]. Such aspects associated with consumer understanding or lack of familiarity can have a negative influence on the willingness to pay for certain attributes [92].

Finally, and regarding possible implications for businesses, it is worth highlighting the interest of designing business strategies that focus on reducing the CF of these products. As consumers value this attribute significantly, this may contribute to increased profits due to consumers’ greater willingness to pay, even with the higher costs associated with CF mitigation.

## 4. Conclusions

Society is currently concerned that food production, and particularly meat production, may contribute to an increase in GHG emissions and, therefore, to climate change. Consumers are not unaware of this and they are questioning meat and meat products more and more for various reasons, convictions, or beliefs. In this context, this study has been undertaken to understand how consumers actually convert their awareness into established purchase choices, and also, into their willingness to pay a premium for certain environmental characteristics of meat.

Consumers point at “Origin” as the main attribute, followed by carbon footprint (CF) and extensive production system. Additionally, attributes “Organic” and “Price” are situated last. In terms of willingness to pay, this is quite variable, subject to consumer profile and society’s sensitisation to climate change. 

The segmentation of consumers we have conducted allowed us to identify three consumer groups with homogeneous preferences. Specifically, consumers have been defined as: “Conscious Consumers” with higher environmentally friendly purchasing habits and attitudes than the others; “Balanced Consumers” with medium willingness to pay for environmental attributes and attitudes; “Indifferent Consumers” with lower willingness to pay and lower environmental attitudes.

Finally, the CF labelling of meat products can help to add value to high-quality, sustainable, and environmentally friendly products. 

Certainly, it would have been preferable to have a bigger and more representative sample of the Spanish population in terms of the other sociodemographic characteristics such as income and academic level, which was difficult to achieve due to the willingness to answer the survey and the study’s resources. Therefore, in order to confirm our findings, new studies in other countries may be useful. 

In addition, in order to either promote these goods or enhance the perception and consumption of products of animal origins, future research should concentrate on enhancing the study of consumer behaviour regarding different product developments and marketing strategies of meat substitutes.

## Figures and Tables

**Table 1:** Sociodemographic profile of the sample (%).

	Variable	Sample (%)
Sex	Male	42.3
	Female	57.7
Age	19–34	24.9
	35–50	27.6
	>50	47.5
Monthly income	<EUR 1200/month	39.9
	EUR 1201–EUR 2400/month	24.6
	EUR 2401–EUR 3600/month	9.6
	>EUR 3600/month	24.2
	DK	1.7
Academic level	No studies	1.6
	Secondary Education/Vocational Training	8.1
	University Degree	90.3

Note: DK: Do not Know.

**Table 2:** Selected attributes.

Attributes/Product	Beef (Filleted Meat)
Production system	Extensive
	Intensive
Origin	Local
	National
	Imported (EU)
Carbon footprint, kg of CO_2_/kg of product	8 kg
	18 kg
	28 kg
Organic	Yes
	No
Price	EUR 10/kg
	EUR 15/kg
	EUR 20/kg

**Table 3:** Results from the logit model (Choice experiment) for the overall sample.

	Overall Sample	
	Coefficient ^a^ (SE) ^b^	z-Value
ASC	−0.1173 (0.1593)	−0.76
Price	−0.0873 (0.0153) ***	−5.76
Extensive	0.4561(0.0633) ***	7.20
Local	1.2516 (0.0857) ***	14.60
National	0.7929 (0.0699) ***	11.34
Footprint 18 kg	−0.5139 (0.0985) ***	−5.21
Footprint 28 kg	−0.5667 (0.0761) ***	−7.39
Organic	0.4916 (0.0773) ***	6.39
Loglikehood	−2085.1404	

^a^ Significance: *** *p* < 0.01; ^b^ Standard Error (SE).

**Table 4:** Description of clusters and the overall sample in terms of attitudes and behaviour towards meat purchase and carbon footprint.

Variable	Cluster 1 (27.9%)	Cluster 2 (39.6%)	Cluster 3 (32.5%)	Overall Sample (N = 362)	Sig. ^a^
Importance given to … when purchasing food (1: not at all important; 5: very important)					
Price	3.10	3.27	3.19	3.23	***
Packaging and Presentation	3.35	3.09	3.08	3.15	***
Origin	4.33	3.54	2.23	3.30	***
Quality labelling (PDO, PGI…)	4.39	3.45	2.26	3.31	***
Impact of the production system on the environment	4.26	2.64	1.51	2.68	***
Local/regional production	4.67	3.76	2.43	3.55	***
Impact on climate change	4.15	2.62	1.49	2.66	***
Place of purchase	4.02	3.53	2.79	3.40	***
Production system and its effect on quality	4.31	3.25	1.90	3.06	***
Meat purchasing frequency 1 (less than once/week), 2 (1–2 times/week), 3 (3 or more times/week)					
Average	2.18	2.52	2.61	2.45	***
Do you know or have heard of carbon footprint? 0 (no), 1 (yes)					
Average	0.76	0.70	0.62	0.69	***
To what extent do you think agriculture and livestock farming have an impact on greenhouse gas emissions and climate change? 1 (don’t know), 2 (little or none), 3 (significant)					
Average	2.59	2.45	2.45	2.46	***
Would you be willing to pay a premium for a food product that was environmentally friendly? 1 (Would not be willing), 2 (May be willing), 3 (Would be willing)					
Average	2.67	2.39	2.18	2.41	***
Would you be willing to change your consumption habits (buying products that are produced more locally in order to reduce transport requirements/ buy products in bulk in order to reduce the use of packaging…) in order to help fight climate change? 0 (no), 1 (yes)					
Average	1.00	0.96	0.90	0.95	***
Awareness of carbon footprint logos (0: unaware; 1: aware of all of them)					
Average	0.85	0.28	0.19	0.39	***

^a^ Significance: *** *p* < 0.01.

**Table 5:** Lifestyles and consumer attitudes towards the environment are scored against a number of statements that fully (7) or not at all (1) reflect their lifestyles.

Var.	Description	Cluster 1	Cluster 2	Cluster 3	Total	Sig. ^a^
SL_1	I try to save energy at home by using efficient electronic appliances, led lights	6.43	6.03	5.88	6.09	***
SL_2	I try to walk or use a bicycle or public transport to move around for shopping or work	5.08	4.07	4.15	4.37	***
SL_3	I try to reduce the use of plastics in my household by using recyclable shopping bags	6.33	5.36	5.36	5.62	***
SL_4	I try to buy local food or food from my region to reduce the transport distance from the production area to the supermarket/store	6.09	5.08	3.85	4.96	***
SL_5	I try to buy more unpackaged or bulk foods to reduce packaging and pollution	5.58	4.58	3.75	4.58	***
SL_6	I try not to buy online (both food and other products) as it has higher environmental impact than physical store shopping, because they have to send the product only to my house and it pollutes more	4.73	4.11	3.41	4.05	***
SL_7	I am interested on food related information because I am concerned about the impact of food on my health	6.10	5.30	4.69	5.32	***
SL_8	I contribute to environmental protection tasks	5.03	3.97	3.30	4.04	***

^a^ Significance: *** *p* < 0.01.

**Table 6:** Results from the logit model (Choice experiment) for the various clusters.

	Cluster 1		Cluster 2		Cluster 3	
	Coefficient ^a^ (SE) ^b^	z-Value	Coefficient ^a^ (SE) ^b^	z-Value	Coefficient ^a^ (SE) ^b^	z-Value
ASC	1.0934 *** (0.3158)	3.46	0.0093 (0.2600)	0.04	−1.0573 (0.2972)	−3.56
Price	−0.0231 (0.0307)	−0.75	−0.0559 (0.0252)	−2.21	−0.1767 (0.0318)	−5.56
Extensive	0.6450 *** (0.1400)	4.61	0.4770 (0.0920)	5.18	0.4888 (0.1339)	3.65
Local	1.4583 *** (0.1797)	8.11	1.4528 (0.1599)	9.08	1.2174 (0.1452)	8.38
National	1.0735 *** (0.1864)	5.76	0.8288 (0.1259)	6.58	0.7369 (0.1042)	7.07
Footprint 18 kg	−0.2927 (0.2277)	−1.29	−0.3405 (0.1469)	−2.32	−0.8278 (0.2070)	−4.00
Footprint 28 kg	−0.7711 *** (0.1559)	−4.94	−0.6850 (0.1352)	−5.07	−0.5390 (0.1272)	−4.24
Ecological	0.6317 *** (0.1927)	3.28	0.5199 (0.1360)	3.82	0.4760 (0.1405)	3.39
Loglikehood	−514.49064		−734.21876		−599.06517	

^a^ Significance: *** *p* < 0.01; ^b^ Standard Error (SE).

**Table 7:** Willingness to pay.

	Overall Sample		Cluster 1		Cluster 2		Cluster 3	
	WTP (Mean)	WTP (Min-Max)	WTP (Mean)	WTP (Min-Max)	WTP (Mean)	WTP (Min-Max)	WTP (Mean)	WTP (Min-Max)
Intensive ⇒ Extensive	5.22	3.34–7.10	n.s.	n.s.	n.s.	n.s.	2.76	1.31–4.21
Imported ⇒ Local	14.33	9.60–19.06	n.s.	n.s.	25.93	4.04−47.88	6.88	4.66−9.11
Imported ⇒ National	9.08	5.39–12.77	n.s.	n.s.	14.81	0.06–29.56	4.16	2.30–6.02
Footprint 8 kg of CO_2e_/kg of meat ⇒ Footprint 18 kg of CO_2e_/kg of meat	−5.88	−7.29– −4.47	n.s.	n.s.	−6.08	−9.92– −2.25	−4.68	−5.90– −3.46
Footprint 8 kg of CO_2e_/kg of meat ⇒ Footprint 28 kg of CO_2e_/kg of meat	−6.49	−8.89– −3.99	n.s.	n.s.	−12.24	−23.58– −0.89	−3.04	−4.44– −1.65
Non organic ⇒ Organic	5.63	4.14–7.11	n.s.	n.s.	9.29	3.05–15.52	2.69	1.71–3.66

Note: n.s.: not significant.

## Data Availability

Data is contained within the article and Supplementary Material.

## Supplementary Materials

The following supporting information can be downloaded at: https://www.mdpi.com/article/10.3390/foods11233899/s1, Figure S1: Representation of the filleted beef used in this study; Table S1: the design of the conducted survey.
