# Supplementary material for: Does Carbon Footprint Play a Relevant Role in Food Consumer Behaviour? A Focus on Spanish Beef
Source: Foods. 2022 Dec 2;11(23):3899. doi: 10.3390/foods11233899 (PMC9738336; doi:10.3390/foods11233899)

foods MDPI

Article

DOES CARBON FOOTPRINT PLAY A RELEVANT ROLE IN FOOD CONSUMER BEHAVIOUR? A FOCUS ON SPANISH BEEF

Supplementary materials:

Table S1. The design of the conducted survey

CONSUMER SURVEY ON CARBON FOOTPRINT LABELING IN FOOD

Consumer habits - Part I

1. Do you purchase the food for your household?

- ☐ Always
- ☐ Most of the time
- ☐ Sometimes
- ☐ Almost never
- ☐ Never (end of survey)

2. Do you consume meat regularly?

- ☐ Yes
- ☐ No

3. How often do you consume meat?

- ☐ Less than once a week
- ☐ 1-2 times per week
- ☐ 3 or more times per week

4. Where do you usually buy meat?

- ☐ Hypermarket/supermarket
- ☐ Local shop
- ☐ Traditional butcher's shop
- ☐ Other: ________________________

5. When purchasing meat, what importance do you attach to each of the following factors (check one option for each factor)?

	Not important	Not very important	Average importance	Quite important	Very important
Price					
Presentation and packaging					
Geographical origin					
Quality labeling (Denomination of Origin (D.O.), Protected					

Geographical Indication (PGI), etc.)					
Impact of the production system on the environment (pollution, depletion of resources, deforestation, etc.), depletion of resources, deforestation...)					
Local/regional production					
Impact of the production system on climate change.					
Place of purchase					
The production system in terms of its impact on product quality (animal welfare, type of feed, etc.).					

6. Has your household's meat consumption been reduced in the last year?
   - ☐ Yes (if yes, indicate in the "other" space why)
   - ☐ No
   - ☐ Other: ___________________________________

7. Do you know, or have you heard of the carbon footprint?
   - ☐ Yes
   - ☐ No

8. What do you relate the carbon footprint to?
   - ☐ Greenhouse gases emitted during the life cycle (from production until it is no longer useful) of a product.
   - ☐ Global warming in general
   - ☐ Green products
   - ☐ Climate change
   - ☐ CO2 offsets

9. Explain in your own words the concept of carbon footprint.

____

10. Do you know, or have you heard about climate change?

- ☐ Yes
- ☐ No

11. Explain in your own words what you understand by climate change.

____

12. How much do you think agriculture and livestock influence greenhouse gas emissions and climate change?

- ☐ A lot
- ☐ A little
- ☐ Not at all
- ☐ Don't know/no answer

Carbon Footprint of Food Production and Climate Change - Part II

Before you continue, please read this information carefully:

Agricultural production and food production and processing systems can be questioned if they are managed in an unsustainable way because the excessive use of resources and the consumption of fossil energies (oil, gas...) can generate significant environmental impacts on the quality of water, soil, air and biodiversity.

To a large extent, these environmental impacts are associated with the emission of greenhouse gases, which are the main responsible for the climate change affecting our planet.

What is the carbon footprint?

The "carbon footprint" is an indicator that quantifies the greenhouse gas emissions generated during the production of a good or service, expressed in kilograms of carbon dioxide (CO2) equivalent. In other words, the carbon footprint tells us how many kilograms of carbon are emitted into the atmosphere when producing one kilogram of product.

For example, in livestock production, this indicator will vary depending on the type of farm and livestock management. Thus, extensive or ecological systems produce fewer greenhouse gas emissions than intensive systems. On the other hand, pasture feeding (reducing the need to buy feed) and less dependence on transport (e.g., if production takes place close to the consumption area) will also reduce greenhouse gas emissions.

13. Have you purchased any food with carbon footprint labeling?

- ☐ Yes
- ☐ No
- ☐ I don´t know

14. Do you remember if you have ever seen the following logos when purchasing food?:

- ☐ Yes
- ☐ No

- ☐ Yes
- ☐ No

- ☐ Yes
- ☐ No

- ☐ Yes
- ☐ No

- ☐ Yes
- ☐ No

- ☐ Yes
- ☐ No

- ☐ Yes

□ No

15. Would you be willing to pay more for an environmentally friendly food product?

□ I wouldn't

□ Maybe I would

□ For sure I would

□ I don't know/I don't care

16. Would you be willing to change your consumption habits (buy products produced closer to your home, to reduce transportation; buy products in bulk to reduce the use of packaging ....) to address climate change?

□ Yes

□ No

Preferences in the purchase of beef and Beef -Part III

Next, suppose you are going to buy beef (1 kg tray of filleted beef) in your usual supermarket or butcher's shop. You can choose between different alternative meats, mainly differentiated by their production system (intensive feedlot; extensive grazing), origin (local/regional, national, import), carbon footprint level (8 kg CO2/kg meat, 18 kg CO2/kg meat, 28 kg CO2/kg meat) with organic or conventional production and price (10 €/kg, 15 €/kg, 20 €/kg).

The carbon footprint of meat is variable, and we can move in a range of 8 to 28 kg of CO2 per kg of meat produced. These values will depend on:

**On the system of exploitation and management of the livestock.

**The self-sufficiency of the farms in animal feeding (grazing - feed).

**And energy consumption (in transport, a car emits about 0.22 kg of CO2 per kilometer traveled, while a truck on average emits 0.66 kg per km).

For example, extensive organic farms based on grazing, with little dependence on feed consumption and close to consumption areas will have lower carbon footprints.

To indicate your preferences, in each of the purchasing situations described below you should choose the meat that best suits your preferences, or, if none of them suits you, check the option "I would not buy any".

Purchasing situation 1.- Choose among the different products (1 kg tray of filleted beef):

	1) Beef from an intensive production system, domestic, with a carbon footprint of 8 kg CO2/kg meat and organic for 20€.	2) Beef from an extensive production system, imported, with a carbon footprint of 18 kg CO2/kg meat and non-organic for 10 €.	3) I wouldn't buy any of them
I would choose:			

Purchasing situation 2.- Choose among the different products (1 kg tray of filleted beef):

	1) Beef produced in an extensive production system, local/regional origin, with a carbon footprint of	2) Beef from an intensive production system, imported, with a carbon footprint of 28 kg CO2/kg meat	3) I wouldn't buy any of them

	28 kg CO_2/kg meat and non-organic for 20€.	and organic for 15€.	
I would choose:			

Purchasing situation 3.- Choose among the different products (1 kg tray of filleted beef):

	1) Beef produced in an intensive production system, imported, with a carbon footprint of 18 kg CO_2/kg meat and organic for 20€.	2) Beef produced in an extensive production system, domestic origin, with a carbon footprint of 8 kg CO_2/kg meat and non-organic for 15€.	3) I wouldn't buy any of them
I would choose:			

Purchasing situation 4.- Choose among the different products (1 kg tray of filleted beef):

	1) Beef produced in an extensive production system, of national origin, with a carbon footprint of 28 kg CO_2/kg meat and organic for 20€.	2) Beef produced in an intensive production system, of local/regional origin, with a carbon footprint of 18 kg CO_2/kg meat and non-organic for 15€.	3) I wouldn't buy any of them
I would choose:			

Purchasing situation 5.- Choose among the different products (1 kg tray of filleted beef):

	1) Beef produced in an intensive production system, domestic origin, with a carbon footprint of 18 kg CO_2/kg meat and non-organic for 10€.	2) Beef produced in an extensive production system, imported, with a carbon footprint of 8 kg CO_2/kg meat and non-organic for 20€.	3) I wouldn't buy any of them
I would choose:			

Purchasing situation 6.- Choose among the different products (1 kg tray of filleted beef):

	1) Beef produced in an intensive production system, imported, with a carbon footprint of 28 kg	2) Beef produced in an extensive production system, local/regional origin, with a carbon footprint of	3) I wouldn't buy any of them

	CO2/kg meat and non-organic for 10€.	18 kg CO2/kg meat and organic for 15€.	
I would choose:			

Socio-demographic characteristics - Part IV

18. Could you please indicate your birth year?

____

19. Gender:

- □ Female
- □ Male

20. Could you tell us the number of people living in your household (including yourself)?

____

21. Could you tell us in what range is the household's net monthly income (including all income)?

- □ < 1.200 € / month
- □ 1.201 - 2.400 € / month
- □ 2.401- 3.600 € / month
- □ > 3.600 € / month

22. Could you please indicate your academic level?

- □ No studies
- □ Primary education
- □ High school/vocational training
- □ University degree

23. Could you please indicate your place of residence?

____

24. Please rate each of the following statements according to whether they are totally adapted (7) or not adapted at all (1) to your lifestyle:

I try to save energy in my home and therefore I use LED bulbs, energy-saving appliances, etc.

not adapted at all □ □ □ □ □ □ □ totally adapted

I try to use public transportation, bicycle or walk for my usual commuting (work, shopping...)

not adapted at all □ □ □ □ □ □ □ totally adapted

I attempt to reduce the use of plastics in my household by using recyclable bags for shopping

not adapted at all □ □ □ □ □ □ □ totally adapted

I try to buy local food or food from my region to reduce the distance that they have to be transported from the production area to the supermarket/store.

not adapted at all □ □ □ □ □ □ □ totally adapted

I try to buy more unpackaged or bulk foods to reduce packaging use and pollution.

not adapted at all □ □ □ □ □ □ □ totally adapted

I try not to purchase online (both food and other products) as it has a greater environmental impact than physical shopping because they have to send the product only to my house and it pollutes more.

not adapted at all □ □ □ □ □ □ □ totally adapted

I am interested in the information related with the food, because I am concerned about the effects of the food on my health

not adapted at all □ □ □ □ □ □ □ totally adapted

I collaborate in tasks of environmental protection

not adapted at all □ □ □ □ □ □ □ totally adapted

Figure S1. Representation of the filleted beef used in this study.

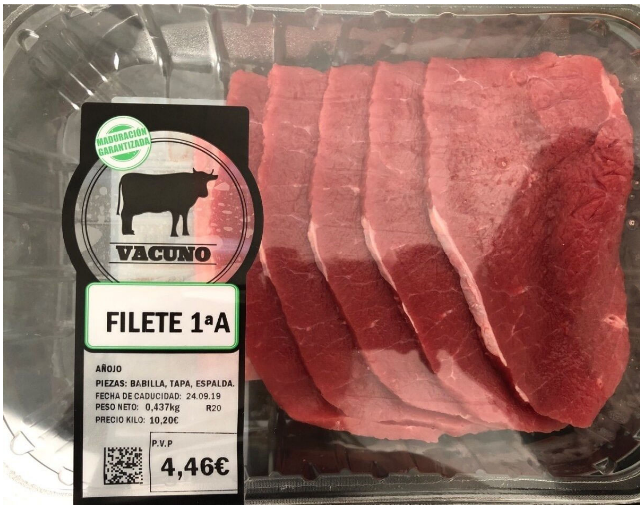

Supplement: Supplementary file 1 [file foods-11-03899-s001.zip › foods-2026907-supplementary.pdf]
